# Inhibition of adaptive therapy tolerance in cancer: is triplet mitochondrial targeting the key?

**DOI:** 10.1002/1878-0261.13406

**Published:** 2023-03-09

**Authors:** Jukka Westermarck

**Affiliations:** ^1^ Turku Bioscience Centre University of Turku and Åbo Akademi University Finland; ^2^ Institute of Biomedicine University of Turku Finland

**Keywords:** acute myeloid leukemia, BH3, glioblastoma, oxidative phosphorylation, protein phosphatase 2A, pyruvate dehydrogenase

## Abstract

Targeted therapies have become a mainstay in the treatment of cancer, but their long‐term efficacy is compromised by acquired drug resistance. Acquired therapy resistance develops via two phases—first through adaptive development of nongenetic drug tolerance, which is followed by stable resistance through the acquisition of genetic mutations. Drug tolerance has been described in practically all clinical cancer treatment contexts, and detectable drug‐tolerant tumors are highly associated with treatment relapse and poor survival. Thereby, novel therapeutic strategies are needed to overcome cancer therapy tolerance. Recent studies have identified a critical role of mitochondrial mechanisms in defining cancer cell sensitivity to targeted therapies and the surprising effects of established cancer therapies on mitochondria. Here, these recent studies are reviewed emphasizing an emerging concept of triplet therapies including three compounds targeting different cancer cell vulnerabilities but including at least one compound that targets the mitochondria. These mitochondria‐targeting triplet therapies have very promising preclinical effects in overcoming cancer therapy tolerance. Potential strategies of how to overcome challenges in the clinical translation of mitochondria‐targeting triplet therapies are also discussed.

## Graphical abstract

Cancer therapies can be effective, but in most cases, cancer cells can develop tolerance against their cell‐killing activities. Recent evidence suggests that targeting cancer cell mitochondria can help in overcoming this therapy tolerance especially when three different drugs are combined with mitochondria‐targeting triplet therapies.

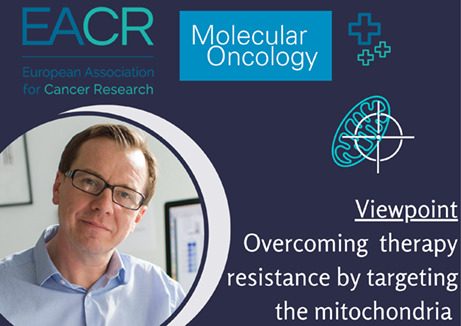

**Abbreviations**
ActD: actinomycin D
AKT: AKT serine/threonine kinase 1
AML: acute myeloid leukemia
AraC: arabinocytosine
AZA: azacitidine
BCL2: BCL2 apoptosis regulator
BH3: BH3‐only proteins
CHOP: cyclophosphamide, vincristine, doxorubicin, prednisone
DRUP: drug rediscovery protocol
ETC: electron transport chain
FOLFIRINOX: 5‐FU, leucovorin, irinotecan, oxaliplatin
GB: glioblastoma
LDH: lactate dehydrogenase
MB: medulloblastoma
MCL1: MCL1 apoptosis regulator
mtDNA: mitochondrial DNA
NPM1c: nucleophosmin 1c
OXPHOS: oxidative phosphorylation
PP2A: protein phosphatase 2A
VEN: venetoclax

Albeit cancer genomes have been sequenced, and genetically mutated drivers have been identified, the promise of a revolution in cancer cures by genome‐guided targeted therapies has only been partly fulfilled [1, 2, 3]. In most cases, this is neither due to the lack of druggability of the driver proteins, nor the lack of therapeutic strategies inhibiting those drivers. Instead, the emerging consensus is that cancers cannot be cured by most of the targeted therapies due to rapidly evolving nongenetic therapy tolerance that allows cancer cells to adapt to inhibition of the driver mechanisms, and thereby avoid apoptosis or other cell death processes [4]. Proposed strategies to overcome nongenetic adaption have included combined targeting of pathways and mechanisms that are induced in response to therapies, or otherwise provide cells with alternative survival signals [5]. Although these combinatorial strategies have increased patient survival in some cases, most patients have yet to relapse and succumb to disease. Thus, the hunt for the optimal therapy combinations based on the parallel targeting approach may never be able to fully solve the most important challenge in cancer therapies, which is therapy‐induced tolerance, and subsequent development of therapy resistance [4].

Are there alternative strategies that could prevent adaptive therapy tolerance? Considering mitochondria as the central cellular machinery involved in the execution of apoptosis via cytochrome C release, and in determining cellular energy balance, it has been previously proposed to be a potential key target for overcoming drug resistance in cancer [2, 6, 7, 8]. Those earlier presumptions are now starting to be validated at least in preclinical therapy models, demonstrating very potent therapeutic effects by drug combinations including mitochondrial targeting. Very interestingly, at least some of these combinations include triplet targeting of mitochondria and signaling pathways, or other driver mechanisms, emphasizing the need for comprehensive co‐targeting of mechanisms behind therapy tolerance to ensure efficient cell death.

Actinomycin D (ActD) is clinically approved for a variety of cancers. Its anticancer effects are traditionally linked to its DNA chelating activities resulting in the inhibition of transcription and DNA replication. De The and collaborators recently discovered a surprising new activity for ActD in targeting Nucleophosmin 1c (NPM1c)‐primed mitochondria in acute myeloid leukemia (AML) cells, resulting in loss of mitochondrial membrane potential, and mtDNA leakage into the cytoplasm [3]. BCL2 inhibitor Venetoclax (VEN) is approved as AML therapy in combination with hypomethylating agents such as Azacitidine (AZA) or Decitabine, or low‐dose Arabinocytosine (AraC). However, like most other combination therapies, a considerable fraction of AML patients also show resistance to these combination therapies [9]. Interestingly, ActD synergized with VEN both in the induction of mitochondria fragmentation and in prolonging mouse survival in a preclinical model of NPM1c‐positive AML [3]. Another recent AML study addressed the contribution of mitochondria on resistance to VEN + AraC [10]. Single‐cell transcriptomics of AML cells resistant to VEN + AraC revealed induction of adaptive resistance associated with increased oxidative phosphorylation (OXPHOS), and activity of mitochondrial electron transport chain (ETC) complex [10]. Importantly, a triplet combination of VEN + AraC with any of the three inhibitors targeting mitochondrial ETC, pyruvate dehydrogenase (promoting pyruvate‐Acetyl CoA conversion), or ClpP protease (degrades misfolded mitochondrial proteins) substantially delayed relapse in their preclinical AML models [10]. An alternative AML triplet therapy combination including mitochondrial targeting was presented recently by Peris and collaborators [11]. In their triplet cocktail, VEN + AZA was combined with small molecule activators of the Protein Phosphatase 2A (PP2A) [12]. PP2A reactivation synergized with VEN by both inhibiting BCL2 phosphorylation directly, and also via ERK‐mediated inhibition of protein stability of another mitochondrial anti‐apoptotic BH3‐family protein, MCL1. The combination of PP2A reactivator molecules significantly potentiated both the apoptotic activity and the *in vivo* therapeutic activity of VEN + AZA in AML models [11]. Importantly, this was shown by using FDA‐approved Fingolimod (FTY720) as the PP2A reactivating molecule [12], opening an interesting opportunity for a drug repurposing clinical trial with VEN + AZA + Fingolimod triplet combination.

These findings highlight the central role of mitochondrial adaptation in the development of therapy tolerance in AML. However, recent studies indicate that malignant brain tumors might also be particularly dependent on mitochondrial mechanisms. A recent elegant example of this was a study by Gyon and collaborators where they demonstrated an important role for mitochondrial adaptation in response to radiation therapy in glioblastoma (GB) [13]. Production of lactate, an end‐product of mitochondrial glycolysis and an important survival factor for GB cells, is catalyzed by lactate dehydrogenase A and B (LDHA and LDHB) proteins. The authors demonstrated that in glioblastoma tissue, LDHA and LDHB are spatially differentially expressed and only a few cells expressed both isoforms [13]. Functionally, lactate production was only inhibited by co‐targeting of both LDHA and B isoforms indicating that in the absence of one isoform, the other isoform can compensate for its activity. Further, highlighting the selective impact of different aspects of mitochondrial metabolism in determining therapy sensitivity, double LDHA and LDHB inhibition resulted in an increase in OXPHOS activity, but in this context, high OXPHOS translated into radiation therapy sensitivity of glioblastoma cells. In an intracranial tumor model, triplet mitochondrial targeting by a combination of genetic LDHA and LDHB inhibition with irradiation had a superior impact on mouse survival [13]. Our own lab's contribution to uncovering the role of mitochondria in cancer therapy tolerance also comes from studies of brain tumor cells. Following previous findings demonstrating potent synthetic lethality in GB cells by multikinase inhibition and PP2A reactivation [14], we recently demonstrated that the critical kinase targets for this synthetic lethal phenotype were AKT and mitochondrial pyruvate dehydrogenase kinases (PDKs) [15]. Across heterogenous GB and medulloblastoma (MB) cell models, none of the combinations of two compounds from AKT inhibitor (AKTi), PDK inhibitor (PDKi), or PP2A reactivator (PP2Aa) induced cell killing across all cell models, whereas this was observed in all cell models with the AKTi + PDKi + PP2Aa triplet therapy. These findings highlighted that triplet targeting is required to overcome both therapy‐induced adaptation but also the heterogeneity of therapy responses across brain tumor cell populations. Mechanistically, PP2A reactivation blocked compensatory OXPHOS activation by PDKi, and induced mitochondrial proton leakage, whereas the combination of AKTi + PDKi caused widespread BH3‐mediated mitochondrial apoptosis priming [15]. The significant therapeutic impact of the orally dosed mitochondrial triplet therapy was demonstrated in both GB and MB intracranial models.

Collectively these studies strongly indicate mitochondrial adaptation as an important mechanism behind nongenetic drug tolerance [3, 7, 8, 10, 11, 13, 15]. Although the combination strategies used across these studies are seemingly different, they do share certain aspects that might be key for their superior activity over the doublet or monotherapies. Firstly, they all include at least one approach that targets mitochondrial metabolism either directly (inhibitors of electron transport chain complex, pyruvate dehydrogenase, PDK, or ClpP protease), or indirectly (VEN or PP2A activators; Fig. 1). Secondly, most of them include BH3 protein targeting directly by VEN or indirectly via PP2A reactivation. Lastly, most combinations include a compound that inhibits cancer cell survival either via signaling pathway inhibition (AKTi) or via chemotherapy (AraC, AZA, or ActD; Fig. 1). At the mechanistic level, compensatory OXPHOS induction and BH3 protein regulation emerged as two interlinked themes related to adaptive mitochondrial therapy tolerance [7, 8, 10, 11, 15]. This was revealed by the results that VEN treatment, in addition to its well‐established function in blocking BCL‐2 [2], inhibits OXPHOS [8, 10], and that pharmacological PP2A reactivation both dephosphorylates BH3 proteins [11] and inhibits OXPHOS [15]. Together with the evidence that ActD targets mitochondria [3], these results indicate that drugs previously described to have selective target activities may be more promiscuous/have a wider reach and also impact mitochondria function. An interesting future question is also whether the superior cell‐killing activity of triplet therapies requires certain specific mitochondrial functions to be altered, or is it simply that three hits are required to efficiently blunt any adaptive mitochondrial rescue strategies induced by mono ‐and doublet combinations?

**Fig. 1:**
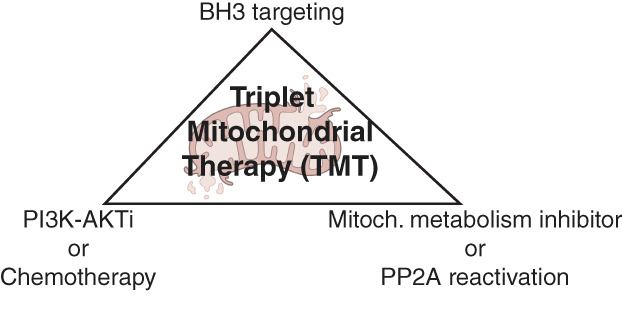
Triplet mitochondrial therapy. The reviewed evidence reveals the therapeutic potential of targeting cancer cells with triplet therapy combinations including one component from each corner of the triangle. Importantly, many current cancer therapies also impact mitochondrial metabolism and/or membrane potential and therefore could be considered as mitochondrial targeting approaches. AKT, AKT serine/threonine kinase 1; BH3, BH3‐only protein; PI3K, phosphoinositol 3 kinase; PP2A, protein phosphatase 2A.

Identifying how to turn these promising preclinical results to the benefit of cancer patients is a crucial question. Considering any higher‐order combination therapies, systemic toxicity and side effects are an obvious concern. On the other hand, current chemotherapy combinations such as CHOP (cyclophosphamide, vincristine, doxorubicin, prednisone) or FOLFIRINOX (5‐FU, leucovorin, irinotecan, oxaliplatin) obviously cause side effects but are yet in routine clinical use due to an acceptable therapeutic window. Similar dose escalation studies that were required for clinical implementation of these quadruplet therapies would obviously be needed for the triplet therapies discussed above. Importantly, most of the drugs used in mitochondrial triplet therapies discussed here have been already used in patients either as approved cancer drugs, in a clinical trial setting, or could be repurposed from other indications such as FTY720. Another potential challenge in clinical testing of the triplet therapies would be organizing a clinical trial combining drugs from different companies and drugs with different patent protection status. Here, clinical drug rediscovery protocols such as DRUP in The Netherlands [16], or IMPRESS in Norway [17], which allow testing of drug combinations using drugs from different associated companies, might provide a solution for the benefit of cancer patients.

## Conflict of interest

The author declare no conflict of interest.
